# Using Video Self-Modelled Social Stories to Teach Social Skills to a Young Child with Autism

**DOI:** 10.1155/2010/834979

**Published:** 2010-06-09

**Authors:** Stacey Litras, Dennis W. Moore, Angelika Anderson

**Affiliations:** Faculty of Education, Monash University, Melbourne 3166, Australia

## Abstract

The present study investigated the effectiveness of combining Social Stories and Video Self-Modelling (VSM) to teach social skills to a three-year-old child with autism. A multiple-baseline across behaviors design revealed that video self-modelled Social Stories were effective at improving all three target behaviors: greeting, inviting to play, and contingent responding. In addition, these behaviors successfully generalized across settings, toys, and communication partners. Concomitant behavior changes, namely, increased levels of communicative behavior and levels of social engagement were also observed. These results support the effectiveness of video self-modelled Social Stories and illustrate the potential of combined intervention techniques for remedying the social deficits faced by this population.

## 1. Introduction

Autism is a neurodevelopmental disorder affecting approximately 60 per 10,000 children worldwide [1, 2]. The isolating nature of this disorder, which results from the core deficits in social interaction and communication, poses significant challenges to the affected population and their families.

Much research has been devoted to develop early intervention strategies targeting the core deficits in autism (e.g., Groden & Cautela (1998); [3, 4]), including social initiations which Koegel and associates have argued may be pivotal to more generalized skill development [5, 6]. Targeting pivotal behaviors for intervention should lead to more widespread beneficial behavior change in untargeted areas [7].

Social Stories and Video Modelling are two relatively novel approaches which appear to hold some promise as emerging intervention strategies. Social Stories [8, 9] are short personalised stories designed to teach children with autism how to manage their behavior during social situations by describing where the activity is likely to take place, when and how it will occur, the emotional perspectives of others involved, and potential responses the target child could display. There is some support for using Social Stories with children with autism for a range of social skills including increasing social initiations and contingent social responding [10], increasing the number of appropriate social interactions during free play [11], increasing socially adaptive behaviors [12], and increasing levels of appropriate social engagement [13]. Some studies have also shown Social Story interventions to produce generalization of acquired skills across settings [10, 13]. Thus although the research base on Social Stories is still quite limited, this intervention strategy appears to hold promise [14].

There has also been considerable research interest in the use of modelling [15], and in particular video modelling, in the treatment of children with autism. Video technology appears well suited to children in general and particularly fitting to the needs of children with autism [16]. Video modelling involves a child watching specifically made video tapes of him or herself, peers or adults engaging in a behavior being taught. The effectiveness of video modelling for children with autism has been demonstrated for a variety of behaviors including increasing conversational speech [17], social initiations, and play-behaviors [18] and play related statements [19]. 

Video-Self Modelling (VSM), made possible through a process called “video feed forward” [20], where editing allows observers to view themselves performing at an advanced level, is thought to be a particularly efficacious use of this technique. Although Sherer et al. [21] reported no difference in the rate of task acquisition as a function of type of model (self or other), others have argued that self-modelling may promote increased self-efficacy, self-confidence, and motivation in the child [22, 23]. 

The effectiveness of VSM has been demonstrated in improving behaviors such as language and social initiations [23], appropriate verbal responses to questions [24], and spontaneous requesting [25]. Incorporating to varying degrees, the explicit use of reinforcement (live reinforcement and embedding within the video), self-management procedures, prompting, explicit instructional rules and multiple exemplars, these interventions have led to rapid and significant gains for participants, with successful generalization across settings in many cases [23–25]. In a recent review of the effectiveness of video modelling interventions including VSM with individuals with autism, Delano [26] concluded that the positive results from the 19 studies reviewed are promising. In the few cases where the intervention was not immediately effective, video-modelling rather than VSM was used and the target behavior was social initiations. Therefore, Delano [26] suggested that when specifically teaching social initiations, VSM might be preferable to video-modelling and additional procedures may need to be incorporated in the intervention.

Social Stories and VSM share several features. Both utilise visual as well as auditory receptive channels, thus capitalising on the relative strengths in visual processing of many individuals with autism. There are obvious differences as well. Scripted Social Stories are highly structured and cue the reader by providing explicit descriptions of the salient elements of the social environment, while VSM is minimally demanding for social interaction and provides opportunity for multiple exemplars/models. A combination of the two involving a real life depiction of a scripted Social Story, thereby capitalising on the strengths of both, could prove an effective intervention for this population. It might be particularly useful for children for whom book reading or story time is aversive.

Several researchers have explored the use of video modelling in combination with Social Stories. Hagiwara and Myles [27] combined electronically presented Social Stories with peer modelling vignettes presented in a computer-based format to teach hand washing and on-task behaviors to three children with autism. Thiemann and Goldstein [10] investigated the combined effects of written text and pictorial cuing with supplemental video feedback on the social communication of five children with autism. Recently Sansosti and Powell-Smith [28] employed a computer-based format to present video modelled Social Stories to increase the social communicative skills of three high-functioning children with autism. Presented as a self-advancing slide show using a computer program, content of the personalised Social Stories was read out by a voice over and then modelled by a similar aged peer. Children viewed their video modelled Social Story once a day in their school setting, immediately before the targeted event occurred (e.g., recess). Overall video modelled Social Stories were effective in improving rates of social communication of participants, though additional social reinforcement and teacher prompting were needed in two cases, and generalization of skills was only observed for one participant. In summary, the VSM/Social Story package may be an effective strategy for teaching social skills to children with autism, one which capitalises on the strengths of both these techniques.

The present study explored the application of a Video Self-Modelled Social Story (VSM Social Story) procedure in teaching a boy with autism three social skills: greeting, inviting to play, and contingent responding to social initiations by others. It was predicted that the presentation of a VSM Social Story containing reinforcement, multiple exemplars, and explicit rules would increase the rate of social initiations in the training setting. It was further hypothesised that social initiations would generalise and be observed outside of the test setting with novel stimuli and situations beyond those specifically modelled. Furthermore, it was hypothesised that increases in social initiations would be accompanied by concomitant behavior changes, namely, increases in levels of verbal/communicative behavior and social engagement/interaction.

## 2. Method

### 2.1. Participant

The participant (pseudonym Jesse) was aged 3 years 5 months at the commencement of training. Independent to this study, Jesse had received a formal developmental assessment, identifying problems with language, socialisation, and repetitive/obsessive behaviours present before age 3, thus fulfilling the criteria for a diagnosis of autism according to the DSM-IV R (Diagnostic and Statistical Manual of the American Psychiatric Association-Fourth Edition Revised). This clinical impression was supported by his score of 34.5 on the Childhood Autism Rating Scale [29], indicating a mild to moderate level of autism. He was recruited from an early intervention center for children with developmental delays and disabilities, based at a university campus.

At time of recruitment, Jesse attended a two-hour program twice a week at the early intervention center and also met with a speech therapist for a two-hour, one-on-one session every fortnight. His verbal skills, specifically labelling and requesting, were reported to have recently improved, but his social skills remained limited. Jesse did not generally display spontaneous vocal social initiations. Observed initiations prior to baseline included vocal requests for items such as a preferred toy or object, requests for help or assistance, nonvocal requests for attention by pointing, and nonvocal requests to play or engage in an activity. Pilot testing revealed that Jesse did not respond well to the reading of trial Social Stories and his parents reported that Jesse had a general aversion to being read to.

### 2.2. Setting

All video presentation sessions were conducted in the family room of Jesse's home. All test sessions were also conducted in Jesse's home, using the exact locations depicted in the videos: the front door, family room, meals area, and kitchen. Generalization probes were obtained through free-time observations both at Jesse's home and at the early intervention center.

### 2.3. Dependent Measures and Data Collection

Two social initiatory behaviors—“greetings” and “inviting to play”—and a third social skill, “contingent responding”, were identified in consultation with the parents and via three 20-minute, free-time observation sessions at both the home and the early intervention center. Specific antecedents to the behaviors, which could serve as visual cues, were identified and a listing of his preferred toys and tasks was also obtained. This information was used to create contextually and environmentally relevant Social Stories and VSM scenes with appropriate reinforcement elements to evoke motivation [30]. 

Dependent measures were the frequency (by opportunity) of the three target behaviors—“greeting”, “inviting to play”, and “contingent responding”. In addition, two concomitant measures, the frequency of vocal/communicative behavior and frequency of social engagement/interaction, were obtained. Behavior specifications for the dependent and concomitant measures are provided in Table 1.

Data were collected by the first author and Jesse's parents. The parents were trained in the observation and recording procedures and also provided with instruction sheets for review as required. Test session data were collected by the first author, with the exception of two specific data points per session, where naturalistic and contextually relevant replication of video scenes for the greeting behavior, namely, “dad coming home from work” and “Jesse coming home with dad to mum”, required parental recording. The parents also recorded anecdotal data including particular reactions to or distractions during the video viewing sessions.

### 2.4. Experimental Design

A single-subject, multiple-baseline across behaviors design was employed to assess the effectiveness of the intervention. The study comprised of three phases: baseline, intervention, and follow up. Each phase was executed consecutively in a time-lagged fashion across the three target behaviors once a stabilised shift in performance was evident for the previous target behavior.

### 2.5. Materials

#### 2.5.1. Social Story Content

Three Social Stories were designed, each addressing one of the three target behaviors and adhering to the recommended Social Story structure of two to five descriptive, perspective and/or affirmative sentences for every directive sentence [9] (The Social Stories are available from the first author on request).

#### 2.5.2. Video Content

Three self-modelled videos were produced, with each video specifically targeting one behavior. As Jesse had an aversion to books, the sentences of the Social Stories were translated into a dialogue between two animated puppets, thereby retaining the structure, meaning, and content of the Social Stories but without the traditional book format. After an initial scene containing the title of the film accompanied by music, each video began with a cartoon animation featuring a puppet tiger and elephant. This animation was constructed using iStop Motion. A real-life representation of the animated Social Story followed, with the participant acting out/modelling the behavior described in the animated Social Story. At the conclusion of each scene, when the desired behavior was displayed, the frame would freeze, and explicit rules reiterating the target behavior were stated in both text and voice-over such as “When I see someone, I say hello”. Introduction of rewards followed, such as text and voice-over saying “WELL DONE!”, “GOOD BOY!”, and “EXCELLENT”, stars, audio cheering, and images of the participant engaging in desired activities. Each video consisted of three examples of the target behavior, thus each video displayed three natural-scenario vignettes, at the conclusion of which reinforcement was provided as described above, followed by a fade-out and 3-second pause (blank screen) leading into the next scene. The “greeting” video for example consisted of: (a) animated Social Story; (b) video modelling scene 1: dad comes home from work and says “hello” to Jesse and Jesse says “hello” back, reinforcement provided (as described above); scene 2: researcher comes through the front door and says “hello” to Jesse and Jesse says “hello” back, reinforcement provided; scene 3: Jesse walks through front door with dad and says “hello” to mum and mum says “hello” back, reinforcement provided. Each video was between three and five minutes long. 

A video recorder, Apple Macintosh computer and toys including a truck, a book, play-doh, and a Mr Potato Head were also required for the construction of the films and throughout test sessions.

### 2.6. Procedure

Prior to any data collection, the project was approved by the Monash University Standing Committee on Ethics in Research involving Humans.

#### 2.6.1. Video Production

Footage for VSM was obtained by using a digital camera to record a series of orchestrated play sessions featuring Jesse and his parents (as communication partners). Scripts were devised and rehearsed with Jesse's parents for each scenario. As vocalisations by the participant were mostly faint and mumbled, recordings of an aged-matched peer saying Jesse's lines were used. The raw footage was edited on an Apple Macintosh laptop using the editing program FinalCut Pro to remove all unwanted behaviors and obvious prompts to produce sequences of the participant correctly performing the target behaviors. The final products were then formatted to three digital video disks (DVD; one for each target behavior) and a copy was issued to the parents.

#### 2.6.2. Observation Procedures

Fifty-minute observation sessions were conducted twice a week, in the early evening at Jesse's home throughout baseline, intervention, and follow-up phases. The first 20 minutes of these sessions included an in vivo reproduction of the scenes featured in the videos, thereby creating three opportunities (directly corresponding to the three scenes depicted in each video) for Jesse to display each target behavior. This generally involved making specific toys or stimuli accessible to Jesse by discretely laying them out within his vicinity. In an effort to allow spontaneous and natural elicitation of the target behaviors, we capitalized where possible on authentic occasions, such as the father's arrival home from work and the researcher's arrival to the home on observation days as opportunities to assess Target Behavior 1—Greeting. (As the elicitation of this behavior was initially relatively weak, verbal prompting was introduced for a series of *four sessions*. This involved a single prompt by parents such as “Jesse, say hello” at the given opportunities. Prompting was not provided for any of the other behaviours). The remaining 30 minutes of the observation session was free-play time during which no further contrived opportunities were created to elicit target behaviors. The entire 50-minute session provided opportunity for Jesse to display generalization of the target behaviors using novel items or stimuli, that is, not those specifically modelled in the videos.

Generalization data was also collected during free-play times at the Early Intervention Centre.

#### 2.6.3. Baseline

Baseline measures were obtained both pre and post video production to account for any incidental learning during the film making process. Baseline procedures entailed the in vivo reproduction of the various scenarios depicted in the videos. Typically this required Jesse being instructed to go to the relevant setting where the specified play materials were laid out and where the communication partner/s then performed their actions.

#### 2.6.4. Intervention, Video presentations

Jesse viewed the target video three times daily at approximately morning, mid-day, and early evening, requiring between three and five minutes per viewing. Upon introduction of each new Target Behavior, Jesse continued to view the videos from the previous phases. All viewing sessions were conducted by Jesse's parents in the family room of their home. Parents used the instructional delivery “It's time to watch your movie” but no further information, prompts, or reinforcement were provided. To ensure adherence to protocol and increase treatment fidelity, parents completed procedural checklists and recorded anecdotal incidents such as distractions or the presence of other people during viewing sessions.

#### 2.6.5. Follow-Up

Follow-up data were obtained 3 weeks after the intervention ended. During this phase no video modelling was provided and data were obtained by conducting test sessions and generalization probes as described above.

#### 2.6.6. Interobserver Reliability

The first author coded and scored all test and generalization sessions. In addition a trained second observer (graduate student) attended and independently observed 30% of the observation sessions in each phase for all three target behaviors. Total agreement reliability was calculated by dividing the lesser frequency by the larger and multiplying by 100%. The mean total agreement reliability was 95% (ranging from 90–100%).

### 2.7. Treatment Fidelity

An advantage of video as a medium for intervention is the capacity it affords for consistency and control of the treatment. In addition treatment fidelity was monitored via a procedural checklist for Jesse's parents including prompting delivery of the verbal instructions, and recording the number of times the video was presented and any anecdotal observations. The researcher observed 10% of video viewing sessions conducted by the participant's parents to ensure the accuracy of protocol adhesion.

### 2.8. Social Validity

Social validity was measured using a parental satisfaction questionnaire on completion of the study. The observed concomitant behavior changes provided further evidence of the social validity of the intervention.

## 3. Results

The data for the target behaviors and concomitant behaviors during VSM training and generalization sessions were plotted. The data for the target behaviors were visually inspected to determine whether a functional relationship existed between VSM training and any observed behavior change. In addition, descriptive data analyses are presented for the associated concomitant behaviors and social validity results.

**Figure 1:**
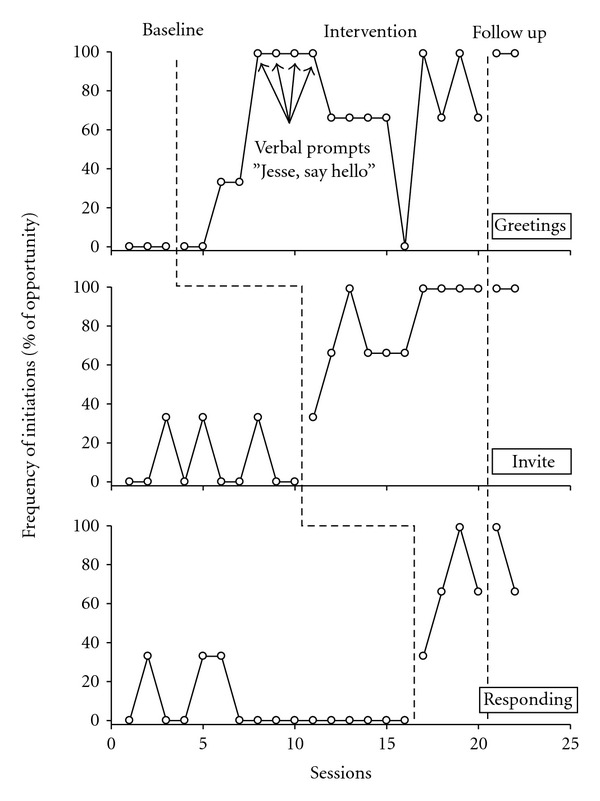
Frequency of scripted social initiations pre-, during and postintervention.

**Figure 2:**
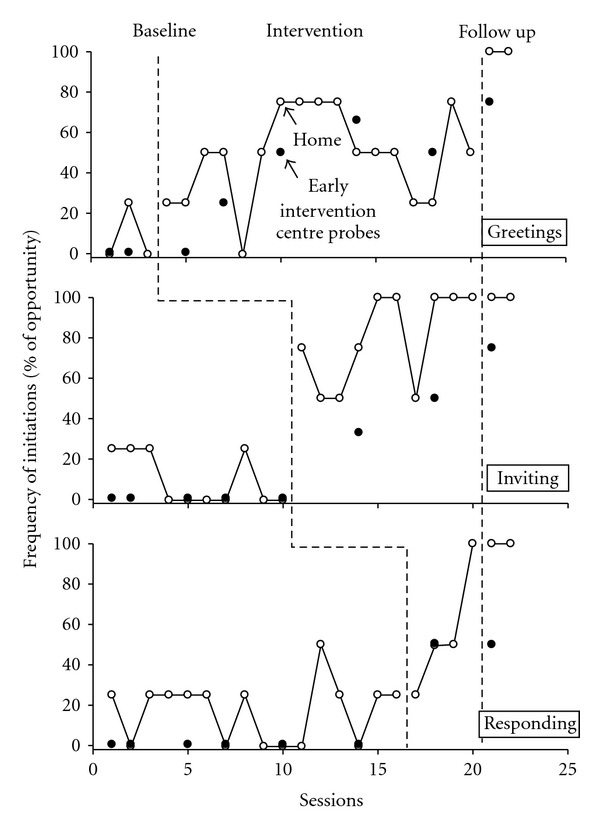
Frequency of generalised social initiations pre-, during and postintervention.

Figures 1 and 2 display the frequency of the three target behaviors, expressed as percentages of opportunities, across baseline, intervention and follow-up phases, for testing data (scripted) and generalization data, respectively.

### 3.1. Greeting

In baseline Jesse engaged in none of the scripted greeting behaviors (Figure 1) in the test settings although he did exhibit one generalized greeting to a grandparent (Figure 2). Following commencement of the intervention, increases were first noticed by the third test session, with 33% (one out of three) successful completion of the target behavior. The introduction of verbal prompting at session 5 was associated with a marked increase of the target behavior to 100%. Test session greetings maintained at between 60% and 100% for the remainder of this phase (phase mean = 62%), and this target behavior was maintained with the participant performing at 100% in follow-up.

Generalization of greetings, both at home and the early intervention center, was exhibited throughout intervention and follow-up phases (see Figure 2). Generalized greeting at the home was seen from the first session in the intervention phase, despite an absence of the behavior in training settings at this time. Home generalizations remained variable throughout the intervention phase (mean = 48.5%). Maximum generalization coincided with the introduction of prompting in testing sessions. In the early intervention center, a steady increase was apparent across the intervention (mean = 37.5%) and follow up (mean = 66%) phases in generalized greetings.

### 3.2. Inviting to Play

During baseline Jesse displayed a fluctuating level of this target behavior with a single invitation to play (33% successful completion) on three occasions in the training setting (mean = 10%) and on four occasions in generalized settings (mean = 10%). Following commencement of the intervention, frequency of the behavior in the test setting increased markedly (mean = 69.7%), and maintained in follow up (mean = 100%). 

Significant generalization of inviting to play was observed at home with mean scores of 9.9%, 79.2%, and 100%, respectively in baseline, intervention, and follow up phases, with no data points overlapping those observed in baseline. In the early intervention center, a steady increase in observed invitations to play were recorded from zero at baseline to a mean of 41.5% during the intervention phase and 75% in follow up.

### 3.3. Contingent Responding

In baseline low rates of contingent responding were observed in both test (mean = 6%) and generalization (mean = 17.2% at home, zero at the early intervention center) settings. Upon implementation of the intervention, a rapid increase in frequency of responding behaviors was evident in the test setting (mean = 66%). This trend was also apparent in the follow up phase (mean = 82%).

Increased generalization of contingent responding relative to baseline was also evident with mean scores of 8.3%, 66%, and 100%, respectively in baseline, intervention, and follow up phases in the home and zero, 50%, and 50% in the equivalent phases in the early intervention center.

### 3.4. Concomitant Behaviors

**Figure 3:**
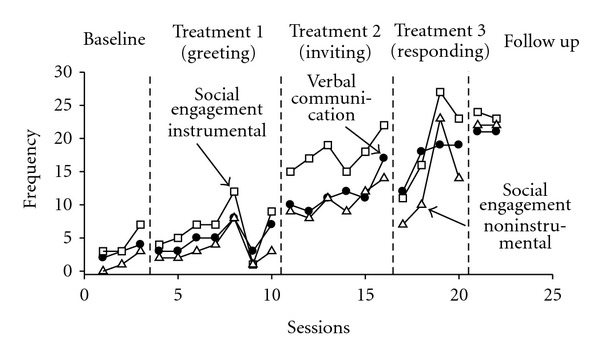
Concomitant behavior changes pre-, during and postintervention.

Figure 3 graphically depicts the concomitant behavior frequency scores (verbal communication, instrumental social engagement, and noninstrumental social engagement). During baseline, Jesse exhibited a relatively low rate of verbal communication and social engagement. Implementation of Treatment 1 (greeting) was associated with slight increases in levels of these behaviors, once again this increase being most apparent with the introduction of verbal prompts. Further increases in all three concomitant behaviors were evident throughout Treatments 2 and 3, with these levels being maintained in the final follow up phase.

### 3.5. Social Validity Results

The parental satisfaction questionnaire and parental interviews assessed the perceived effectiveness of the intervention in teaching Jesse to greet, invite to play, and respond contingently. The parents' responses indicated that they perceived acceleration in Jesse's communicative development and in overall social functioning and general behavior across the intervention period. In addition, the parents reported specifically on qualitative changes in Jesse's execution of the target behaviors over time, becoming less mechanical and more genuine. Particular improvements in the quality of target behaviors were evident at follow-up with elimination of direct copying of the language used in the videos and with Jesse displaying each target behavior accurately, in his natural speaking voice, in the correct social context and with the absence of other verbalisations from the videos. Improved quality of the behaviors included specifications added to invitations to play, such as accurately identifying (naming) who he was inviting and what he actually wanted to play with, for example “Stacey, let's play ball”.

Anecdotal observations also revealed the emergence of spontaneous farewelling behavior by the participant. Jesse began to exhibit “goodbye” behaviors independently and consistently from approximately session 10, through to the end of the follow up phase. Parents reported similar occurrences in encounters with others.

## 4. Discussion

The current study explored the effects of using video self modelled Social Stories to teach three social skills to a young child with autism. The results support the hypothesis that presentation of VSM using the structure of Social Stories, and containing reinforcement, multiple exemplars, and explicit rules would increase the rate of social initiations displayed by the child. As anticipated, the results also showed that social initiations generalized and were observed outside of the test setting and with novel stimuli. The effectiveness of the intervention was demonstrated across three social behaviors: greeting, inviting to play, and contingent responding. Furthermore, the data support the prediction that increases in social initiations would be accompanied by concomitant behavior changes, namely, increases in levels of vocal communicative behavior and social engagement/interaction.

These findings are in accord with previous research demonstrating a positive effect of using Social Stories in teaching greetings [31], initiating comments and contingent responses [10, 13], and video modelling in teaching-related behaviors including conversation skills [17], play-related statements [19], and inviting to play [18].

Furthermore, the rapid acquisition of the target behaviors noted in the present study is consistent with previous findings on the effects of VSM in teaching social skills to children with autism [23–25]. With the exception of “greeting”, increased target skills were displayed following the very first video viewing. The initial delay in eliciting greetings may be due to the noninstrumental nature of this behavior relative to either inviting or contingent responding. Nonetheless, following the addition of prompting, greeting behaviors also improved to a level comparable to the other target skills.

Sansosti and Powell-Smith [28] also combined video modelling and Social Stories to teach “joining in” and “maintaining conversation” behaviors to three children with high functioning autism. For two participants in their study, continual teacher prompting and social reinforcement via child confederates were needed for these behaviors to be displayed. In addition, generalization of acquired skills was only found with one child. Other researchers have reported similar difficulties with video modelling including the necessity of adding live contingencies to video modelling methods in order to improve skill acquisition [32] and lack of generalized effects in the absence of direct training or programming contingencies [11, 12, 19].

Although prompting was also introduced in the present study to strengthen greeting behaviors, prompts were only offered briefly and, upon their withdrawal, the desired behavior was maintained, including in follow-up. In addition, the present study resulted in generalization across all three target behaviors. Methodological comparison of the video content used by Sansosti and Powell-Smith [28] and the present study reveals several variations which may account for these differences. Sansosti and Powell-Smith did not apply any reinforcement principles *within* the video modelled Social Stories, whilst the present study provided verbal, visual and auditory reinforcement in the forms of praise, depictions of the participant engaged in enjoyable tasks, stars, audio cheering, and other musical sound effects. Moreover, Sansosti and Powell-Smith [28] appear not to have provided multiple examples of the targeted behaviors in the peer modelled videos, whereas three variant scenes for each self-modelled target behavior were incorporated in the present study. Further research on the possible benefits of inclusions such as explicit rules [32], embedded reinforcement [23] and multiple exemplars [33], as were utilised in this study, appears justified.

The observed trends in the concomitant behaviours in the current study—though not sufficient to demonstrate a functional relationship—support previous findings that targeting social initiations can lead to collateral improvements in other, nontargeted areas of functioning [6, 7]. The anecdotally reported development of spontaneous “farewelling” behaviors was an unanticipated outcome of the current study. Though not formally observed, the parents' obvious surprise at the emergence of this behavior was indicative of its novelty. Not previously reported, this is an intriguing result for an intervention of this kind. The apparently spontaneous occurrence of farewelling behavior may represent a generalization of greeting behavior based on the premise that both these skills belong to the same response class. Others [5, 10] have suggested that treating social behaviors within a similar response class will ultimately lead to positive changes in related but untreated behaviors.

Though time limitations constrained both the frequency and duration of our postintervention maintenance-assessment, all three target behaviors and the concomitant behaviors were successfully maintained at three-week follow-up, with increased frequency and quality of performance compared to the intervention phase. Anecdotal observations during the intervention indicated that initially Jesse often displayed target behaviors using the exact same language and vocal intonations as were presented in the videos including other verbalisations such as communication partners' lines and verbal reinforcements presented in the video. At follow-up, however, marked qualitative improvements were evident, strengthening evidence for the social validity of the outcomes of the study.

This study contributes to the growing research corpus regarding the effectiveness of technically enhanced video self modelling and the development of evidence-based practices by demonstrating the effectiveness of combined intervention strategies and supporting clinical recommendations for using Social Story interventions in combination with other methods to teach social skills to children with autism [34–37]. Presented via television, this study uniquely contributes to novel methods of implementing visually mediated interventions of this kind and also demonstrates how intervention packages can be applied and evaluated under naturalistic conditions. Video implemented interventions present an unobtrusive teaching method and an activity enjoyed by most participants [22, 23, 38]. With the continuing development of increasingly accessible software for home production of video, requiring minimal technical expertise, VSM interventions can now be easily implemented and readily employed by teachers, parents, or practitioners in naturalistic settings including the mainstream school or the home.

As is true of all *N* = 1 within subject designs, the external validity and generalizability of these results can only be established through replication. Of particular import in this instance is the degree to which existing attending and imitation skills of the child with autism are required; this teaching technique may only be effective for children with autism who demonstrate these skills. Further research establishing the generalization boundaries here, and possible strategies for teaching such attending and imitation skills when not present would be of value, as would systematic replications varying treatment intensity (duration and exposure frequency) when working with children with more severe deficits.

In summary, this study investigated the effects of combining two recently developed and as yet underresearched procedures for remedying the social deficits of a young child with autism. Video self-modelled Social Stories stand as a promising intervention, being straightforward and efficient to implement with possible applications across a wide array of behaviors. This package presents a tool appropriate to autism as it maximizes visual strengths and accounts for attention weaknesses, while also remaining an enjoyable and motivating activity for children. Further research is needed to determine the replicability of the present findings and possible limitations of the procedure.

## Figures and Tables

**Table 1:** Definitions of Dependant Measures.

Social skill	Definition
Greeting	Within 2 seconds of an arrival, child acknowledges a new person with a front facing orientation and with focus directed toward him/her, with a vocal utterance including “hello” or “hi”.

Making invitation to play	Child uses one or more intelligible phrases while positioned within a meter of a peer or adult and with body oriented toward peer or adult to express desire for them to play with him/her (e.g., “come and play”).

Contingent response	Child responds appropriately (vocally or nonvocally) to a peer or adult's utterance within a 3-second interval, through (a) acknowledging (e.g., “hmmm”), (b) agreeing (e.g., head nod, “yeah”), (c) answering a question, (d) responding with a related comment about observable objects or events within the ongoing activity, (e) confirming or clarifying a question or comment from the peer or adult (e.g., “what did you say?”)

Verbal/communicative behaviour	Child makes an intelligible, vocal utterance clearly directed to an adult or to a peer (*not* including responding to other's utterances).

Social engagement/interaction	(1) Child is engaged or interacting with another person for at least 10 seconds, including self-initiated interactions and other initiated interactions; (2) a subset of (1) where child is engaged or interacting with another person for at least 10 seconds *not* in an instrumental capacity (i.e., where child is not interacting for the sole purpose of attaining assistance, attention, etc.)

## References

[1] Fombonne E (2005). The changing epidemiology of autism. *Journal of Applied Research in Intellectual Disabilities*.

[2] Autism Spectrum Australia What is Autism?. http://autismspectrum.org.au/a2i1i1l237l113/what-is-autism.htm.

[3] Koegel LK, Koegel RL, Hurley C, Frea WD (1992). Improving social skills and disruptive behavior in children with autism through self-management. *Journal of Applied Behavior Analysis*.

[4] Konstantareas MM (2006). Social skills training in high functioning autism and Asperger’s disorder. *Hellenic Journal of Psychology*.

[5] Koegel RI, Frea WD (1993). Treatment of social behavior in autism through the modification of pivotal social skills. *Journal of Applied Behavior Analysis*.

[6] Koegel RL, Koegel LK, McNerney EK (2001). Pivotal areas in intervention for autism. *Journal of Clinical Child and Adolescent Psychology*.

[7] Koegel RL, Camarata S, Koegel LK, Matson JL (1994). Aggression and noncompliance: behavior modification through naturalistic language remediation. *Autism in Children and Adults: Etiology, Assessment and Intervention*.

[8] Gray CA Social stories and comic strip conversations: unique methods to improve social understanding.

[9] Gray CA, Garrand JD (1993). Social stories: improving responses of students with autism with accurate social information. *Focus on Autistic Behavior*.

[10] Thiemann KS, Goldstein H (2001). Social stories, written text cues, and video feedback: effects on social communication of children with autism. *Journal of Applied Behavior Analysis*.

[11] Scattone D, Tingstorm DH, Wilczynski SM (2006). Increasing appropriate social interactions of children with autism spectrum disorders using social stories. *Focus on Autism and Other Developmental Disabilities*.

[12] Sansosti FJ, Powell-Smith KA (2006). Using social stories to improve the social behavior of children with Asperger syndrome. *Journal of Positive Behavior Interventions*.

[13] Delano M, Snell ME (2006). The effects of social stories on the social engagement of children with autism. *Journal of Positive Behavior Interventions*.

[14] Sansosti FJ, Powell-Smith KA, Kincaid D (2004). A research synthesis of social story interventions for children with autism spectrum disorders. *Focus on Autism and Other Developmental Disabilities*.

[15] Bandura A (1969). *Principles of Behavior Modification*.

[16] Sturmey P (2003). Video technology and persons with autism and other developmental disabilities: an emerging technology for PBS. *Journal of Positive Behavior Interventions*.

[17] Charlop MH, Milstein JP (1989). Teaching autistic children conversational speech using video modeling. *Journal of Applied Behavior Analysis*.

[18] Nikopoulos CK, Keenan M (2004). Effects of video modeling on social initiations by children with autism. *Journal of Applied Behavior Analysis*.

[19] Taylor BA, Levin L, Jasper S (1999). Increasing play-related statements in children with autism toward their siblings: effects of video modelling. *Journal of Development and Physical Disabilities*.

[20] Hitchcock CH, Dowrick PW, Prater MA (2003). Video self-modeling intervention in school-based settings: a review. *Remedial and Special Education*.

[21] Sherer M, Pierce KL, Paredes S, Kisacky KL, Ingersoll B, Schreibman L (2001). Enhancing conversation skills in children with autism via video technology: which is better, ‘self’ or ‘other’ as a model?. *Behavior Modification*.

[22] Buggey T (1999). Look! I’m on TV!. *Teaching Exceptional Children*.

[23] Buggey T (2005). Video self-modelling applications with students with autism spectrum disorder in a small private school setting. *Focus on Autism and Other Developmental Disabilities*.

[24] Buggey T, Toombs K, Gardener P, Cervetti M (1999). Training responding behaviors in students with autism: using videotaped self-modelling. *Journal of Positive Behavior Interventions*.

[25] Wert BY, Neisworth JT (2003). Effects of video self-modelling on spontaneous requesting in children with autism. *Journal of Positive Behavior Interventions*.

[26] Delano ME (2007). Video modeling interventions for individuals with autism. *Remedial and Special Education*.

[27] Hagiwara T, Myles BS (1999). A multimedia social story intervention: teaching skills to children with autism. *Focus on Autism and Other Developmental Disabilities*.

[28] Sansosti FJ, Powell-Smith KA (2008). Using computer-presented social stories and video models to increase the social communication skills of children with high-functioning autism spectrum disorders. *Journal of Positive Behavior Interventions*.

[29] Schopler E, Reichler RJ, Rochen Renner B (1988). *The Childhood Autism Rating Scale (CARS) for Diagnostic Screening and Classification of Autism*.

[30] Koegel RL, Koegel LK (1995). *Teaching Children with Autism: Strategies for Initiating Positive Interactions and Improving Learning Opportunities*.

[31] Swaggart BL, Gagnon E, Bock SJ, Earles TL, Quinn C, Myles BS (1995). Using social stories to teach social and behavioral skills to children with autism. *Focus on Autistic Behavior*.

[32] Apple AL, Billingsley F, Schwartz IS (2005). Effects of video modeling alone and with self-management on compliment-giving behaviors of children with high-functioning ASD. *Journal of Positive Behavior Interventions*.

[33] Stokes TF, Baer DM (1977). An implicit technology of generalization. *Journal of Applied Behavior Analysis*.

[34] Attwood T (2000). Strategies for improving the social integration of children with Asperger syndrome. *Autism*.

[35] Gray CA, Schopler E, Mesibov GB, Kunce LJ (1998). Social stories and comic strip conversations with students with Asperger syndrome and high-functioning autism. *Asperger Syndrome or High-Functioning Autism?*.

[36] Rogers SJ (2000). Interventions that facilitate socialization in children with autism. *Journal of Autism and Developmental Disorders*.

[37] Safran SP (2001). Asperger syndrome: the emerging challenge to special education. *Exceptional Children*.

[38] Charlop-Christy MH, Le L, Freeman KA (2000). A comparison of video modeling with in vivo modeling for teaching children with autism. *Journal of Autism and Developmental Disorders*.

